# Farmer decision making for hybrid maize seed purchases: Effects of brand loyalty, price discounts and product information

**DOI:** 10.1016/j.agsy.2024.104002

**Published:** 2024-06

**Authors:** Pieter Rutsaert, Jason Donovan, Mike Murphy, Vivian Hoffmann

**Affiliations:** a International Maize and Wheat Improvement Centre (CIMMYT), Nairobi, Kenya; b International Maize and Wheat Improvement Centre (CIMMYT), Texcoco, Mexico; c International Food Policy Research Institute (IFPRI), Washington, DC, USA; d Carleton University, Ottawa, ON, Canada

**Keywords:** Kenya, Varietal turnover, Food security, Agro-dealers, Seed systems

## Abstract

**CONTEXT:**

Each year public and private sector maize breeding programs in Kenya deliver high-yielding hybrids that are resistant to drought, pests, and diseases. Yet, most Kenyan maize farmers purchase older, well-known hybrids. While the ‘varietal turnover’ problem is well known, few solutions have emerged.

**OBJECTIVE:**

The potential for seed companies and retailers to influence farmers' product selection towards new products remains an open question. In-store marketing that induces farmers to experiment with new products may be a scalable and cost-effective way to advance seed systems development.

**METHODS:**

Our controlled field experiment with 600 farmers in Kenya comprised a mock agrodealer store stocked with locally available hybrids, where half the farmers who participated faced an out-of-stock situation for their preferred product. The influence of price promotions and product performance information on farmers' seed choice were assessed.

**RESULTS AND CONCLUSIONS:**

When a participant's preferred product was available, performance information and discounts had no effect on decisions. However, when the preferred product was unavailable, the treatments had limited effects on product selection. Prior experience and brand loyalty stood out as the strongest predictors of seed product selection.

**SIGNIFICANCE:**

Our work explored the potential for two interventions—information and price discounts—to influence farmers' product selection. While these interventions showed limited influence on selection, the study design provides a clear starting point for future related experiments. More public and private investments are required to generate timely, comparable, and reliable information on seed performance. The strong effect of brand loyalty favors larger-sized seed companies with sizable marketing budgets.

## Highlights

• Maize farmers in Kenya purchase hybrids released 20 or more years ago, despite availability of new higher-performing hybrids.
• We conducted an experiment to assess farmers' purchase decisions when their preferred variety was available vs out-of-stock.
• Virtually no experimentation was done with new seed products when product typically purchased was in-stock.
• Without access to their preferred variety, brand loyalty and prior experience stood out as predictors of seed selection.
• Public and private investments are required to generate timely, comparable and reliable information on seed performance.

## Graphical abstract

**Figure:**
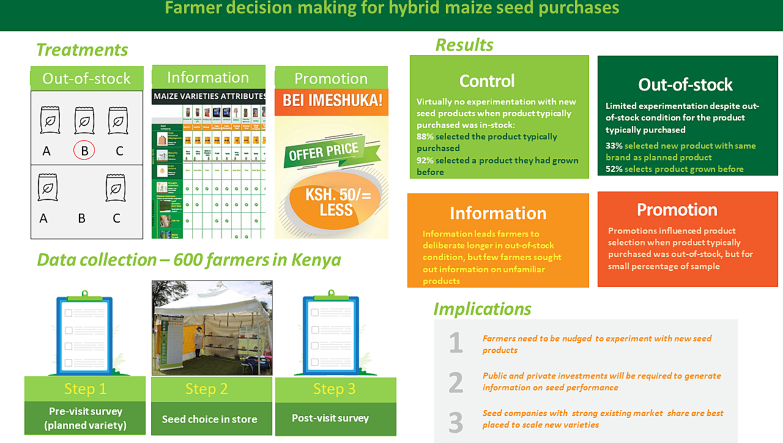

## 1 Introduction

Since the Green Revolution of the late 1960s and 1970s, the development and delivery of new hybrids and improved varieties have been a central element in government and donor strategies to support increased smallholder productivity and more affordable food supplies for urban populations. Population growth and urbanization will continue to put pressure on smallholder agriculture to provide greater volumes of nutritious foods (Giller, 2020). The implications of climate change, including the increased burden of biotic and abiotic stresses on crop production, magnify these pressures. Therefore, it is of critical importance that new climate-resilient and more nutritious hybrids and varieties be developed and that these replace the older hybrids and varieties that still dominate the seed market for most crops and regions (Abate et al., 2017; Rutsaert and Donovan, 2020a; Thiele et al., 2020; Walker and Alwang, 2015). Among the crop breeding efforts in Sub-Saharan Africa that donors and government have prioritized is maize—a crop that is critical for food security and income generation for smallholders.

In East Africa, the lion's share of public investments in maize breeding has focused on the development of hybrids with increased drought and disease tolerance (Atlin et al., 2017; Prasanna et al., 2021). Product development and delivery has generally been left to (quasi-) private sector actors, motivated by farmers' willingness to purchase hybrids on an annual basis as well by the relatively low production and distribution cost of maize seed (Donovan et al., 2021; Spielman et al., 2010; Spielman and Kennedy, 2016). In general, Kenyan farmers have access to hybrid maize seed products^1^ during the long and short maize growing seasons from dozens of domestic and international seed companies that operate in the country. Farmers choose between several newly released climate-resilient hybrids and dozens of older, well-known hybrids, with agro-dealers regularly introducing new seed products (Rutsaert and Donovan, 2020b). The use of maize hybrids by smallholders has contributed to higher incomes and the building of asset endowments (Mathenge et al., 2014). While the parastatal Kenyan Seed Company (KSC) retains a large market share in certain maize-growing regions, smaller privately-owned seed companies have emerged over the past two decades (Langyintuo et al., 2010) and captured market share in the mid and low altitude growing regions. Hybrid maize is sold across the roughly 5000 agro-dealers that operate in the country (Mabaya et al., 2021).

A key assumption that underpins public and donor investments in crop breeding is that farmers will replace old hybrids and varieties when they become aware of the superiority of new planting material (CGIAR, 2018). However, this assumption has not held in the case of hybrid maize in Kenya. The average weighted age of hybrids in Kenya is estimated between 15 and 20 years (Abate et al., 2017; Rutsaert and Donovan, 2020b; Smale and Olwande, 2014). The dominance of old maize hybrids in the Kenyan market has emerged as an obstacle to scaling up the use of more resilient and productive hybrids (Abate et al., 2017; de Groote et al., 2015; Rutsaert and Donovan, 2020b; Chivasa et al., 2022). Following the categorization of Walker and Alwang (2015), we consider products that were released before 2002 (now 20 years ago) as ‘old’ hybrids. The same authors concluded that a weighted (by market share) average product age of 10 years or below signals strong advancements in plant breeding from an economic perspective and this has been adopted as the target for CGIAR plant breeding efforts (CGIAR, 2021). For example, Duma 43 is an old and widely sold early maturity hybrid in Kenya (de Groote et al., 2015; Rutsaert and Donovan, 2020b) and a benchmark for testing the performance of potential future products. Rezende et al. (2020) showed how most new hybrids outperformed Duma 43 in yield under optimal and drought stress conditions.

Local seed companies face a short time period ahead of each growing season to sell their hybrid seed stock, and thus depend on agro-dealers to distribute products quickly to farmers. Within this context important questions arise related to how farmers make seed purchase decisions and the extent to which these decisions could be influenced at the point-of-sale by seed companies and retailers. Marketing efforts by seed companies typically seek to show farmers the performance of new products in fields through demonstration plots and farmer field days (MacRobert, 2009; Makinde and Muhhuku, 2017; Misiko, 2013). While these can be effective at informing farmers about the advantages of a new product (Emerick and Dar, 2020), they are expensive to carry out, requiring investments in monitoring, input provision, and farmer recruitment. Moreover, when multiple companies promote different products in the same area, the benefit to any one company of doing so decreases. In a recent study, only a small fraction (1%) of Kenyan farmers who purchased a new hybrid at an agro-dealer claimed that their purchase decision had been influenced by a demonstration plot or field day (Rutsaert and Donovan, 2020b). In India, Stone (2007) found that cotton farmers attending demonstration plots recalled the farmers who grew or promoted the seed product, but rarely recalled the agronomic details of the products showcased.

The extent to which maize seed companies can build market share for newly released hybrids, while simultaneously removing older hybrids from the market, has rarely been discussed. The nature and depth of the relationship between seed companies and agro-dealers may be critical to getting new hybrids to more farmers. Among the defining principles of integrated seed systems development approach advanced by Louwaars and de Boef (2012), Thujssen et al. (2015), and others are the recognition of the structure of seed value chains, the potential to promote entrepreneurship and market orientation, and a call to support an enabling environment for seed value chain development. Addressing the challenges of low varietal adoption and slow turnover will require new insights on the demand-side drivers of seed value chain development, to include insights on how farmers take their seed product purchase decisions and the risks inherent in the marketing of new seed products faced by seed companies and retailers (Donovan et al., 2021).

Collaboration between seed companies and agro-dealers in the form of in-store marketing may induce farmers to consider and experiment with new products. In-store marketing may offer a scalable and cost-effective way to influence farmers' seed choice in private-sector dominated seed systems. Discussions on product innovation have pointed out the potential for product branding and labeling to shape consumers perceptions of quality (Caswell and Mojduszka, 1996; Fernqvist and Ekelund, 2014; Srinivasan and Till, 2002). The potential for price discounts combined with information treatments (nudging) to influence consumer choice has been explored extensively in the context of human diets and nutrition (Ball et al., 2015; Brimblecombe et al., 2017; Magnus et al., 2016). Potential options available in the retail space to influence choice are brand (as a signal of quality), price (higher prices as a signal of quality or lower prices offering costs savings), and information (e.g., on-farm performance analysis). No other study, to the best of our knowledge, has examined the effect of information or price discounts on farmer seed choice in the retail environment.

This article presents the results of a controlled field experiment in which farmers purchased a bag of maize seed in a mock agro-dealer store. Half of the sample faced an ‘out-of-stock’ (OOS) condition for their preferred product and therefore had to select a new one. To better understand the ‘stickiness’ of farmers' seed choices, despite continuous improvement in the performance of new hybrid maize products, we tested the effects on purchase behavior of in-store information on product attributes, price-based promotion, and brand loyalty, both in a situation where farmers do and do not have access to their product of choice. We employed an experimental design that allowed us to observe farmer behavior in a natural setting by mimicking the environment in which seed choices are made. Similar approaches have been used to understand consumer behavior in supermarkets (Verbeke et al., 1998), food choice in cafeterias (Carins et al., 2017), and demand for food safety (Birol et al., 2015; Hoffmann et al., 2021). While the mock agro-dealer store used in this experiment was admittedly not a *perfectly* natural setting, it was designed to exhibit key features of the shops in from which farmers in Kenya buy seed, including several seed options from different brands, no to little comparable information and a lack of attribute-specific labeling. To the best of our knowledge, no other research on seed systems has replicated a retail environment to understand how farmers take decisions on seed products, although similar approaches have been applied in other areas of research (e.g. Areni and Kim, 1994; Petermans et al., 2021; Schnack et al., 2020).

The next section draws insights from the seed systems and marketing literatures on product selection and factors that influence product selection. Section three explains the OOS experiment carried out in Kenya to test farmers' seed selection process when their preferred product was available or not available. Section four presents the results. The fifth section discusses the implications of the experiment for future research on formal seed systems and options for speeding varietal turnover, followed by the conclusion.

## 2 Product selection and farmer decision-making

The marketing literature recognizes that influencing consumer choice requires direct or indirect contact with a brand at different moments and through a variety of channels, often referred to as ‘touch-points’ (Baxendale et al., 2015; Court et al., 2009). In the case of seed, touch-points include roadside demonstration plots, radio and television advertisements, agricultural shows and field days, sales agent visits to farmer groups, and farmer engagement at the store during the seed sales season. The retail environment is considered as one of the more influential touchpoints (Baxendale et al., 2015).

However, the notion of agro-dealers as a touchpoint for shaping farmers' choices on input decisions has received limited attention. Once exception was work by Dar et al. (2021) who showed that in the Indian state of Odisha, targeting input suppliers with information interventions was 50% more effective at generating farmer adoption of a new rice seed product compared to conventional activities (seed packs, demonstration plots, field days) conducted by the government extension service.

One key factor that shapes in-store decision-making is customers' brand perception. Hoyer (1984) described that brand loyalty resulted from post-purchase product performance and the expectation that a product from the same brand will perform similarly. Brand loyalty is critical to commercial success as retaining customers often requires less resources than recruiting new ones (Knox and Walker, 2001).

In the case of maize seed in Kenya, where most seed is sold in 2 kg-sized branded bags, farmers only have one or two seasons per year to test a new brand. Once brand loyalty is established, opportunities for this to be challenged are limited, and it is likely to persist unless a negative signal, such as a crop failure, occurs.

Price is another important driver of product choice which can be leveraged at the retail level. Price promotions have been one of the most successful marketing strategies used by companies to attract new customers or move existing customers between a companies' brands (Blattberg et al., 1995; Kumar and Leone, 1988; Voss and Seiders, 2003). This strategy encourages customers to experiment with a new product by reducing the cost of failure of that product (Heiman and Hildebrandt, 2018). Research on price promotions has shown that they harnesses consumers' intuitive decision-making at the point of purchase (Samson and Voyer, 2012).

Hoffmann et al. (2021) found that price promotions had an immediate impact on sales of maize flour in Kenya labeled as tested for aflatoxin, and that this effect persisted for several weeks after the promotion ended, but then faded. The effect of in-store price promotions has not been tested on seed product sales.

Advertising and information can also be used to influence farmers at the point of sale. The aim of this strategy is to provide information about the performance of the product (Heiman and Hildebrandt, 2018). Based on an analysis of cross-sectional data, Simtowe et al. (2019) find that information about product performance has the potential to significantly increase adoption of drought-tolerant maize products in Uganda. An evaluation of the store environment of agro-dealers in Kenya showed that the use of marketing materials or detailed product information was limited and that most stores did not make seed prices visible (Rutsaert et al., 2021). As noted in the introduction, comparable information on varietal performance is not available to farmers.

The impact of stockouts on both consumer choice and retail sales has been thoroughly explored for fast moving consumer goods (Fitzsimons, 2000; Helm and Stölzle, 2007; Sloot et al., 2005). A key question for brands is whether customers facing a stockout will delay their purchase, go to a different store (negatively affecting the retailer), select another product from the same brand, or switch to a different brand (negatively affecting the brand).

## 3 Material and methods

### 3.1 Sample selection

**Fig. 1:**
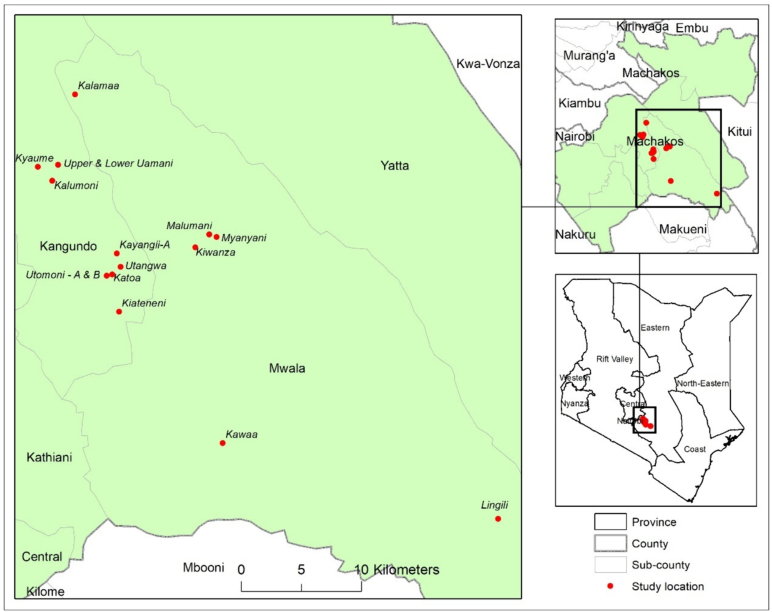
Locations of agro-dealer surveys and farmer intercept interviews.

The study was conducted in Machakos County, Kenya, from late February to late March 2021, just before the start of the main (long) maize planting season.^2^ Three of the eight sub-counties of Machakos were randomly selected, namely, Mwala, Matungulu and Kangundo. Villages were stratified by sub-county and 20 were randomly selected: seven villages in Mwala and Matungulu and six villages in Kangundo (Fig. 1). Farmers' lists were obtained from all the selected villages, and farmers who met the following selection criteria were retained in the sampling frame: (i) the participant had purchased hybrid maize seed at least once in the last 3 years and (ii) the participant reported to be involved in household decision-making about maize seed purchases. The sample was stratified by gender, and a total of up to 32 farmers were selected from each village, balanced by gender to the extent feasible.^3^ Overall, men had a higher percentage of no-show leading to a higher percentage of women participating in the study.

### 3.2 Study design

**Fig. 2:**
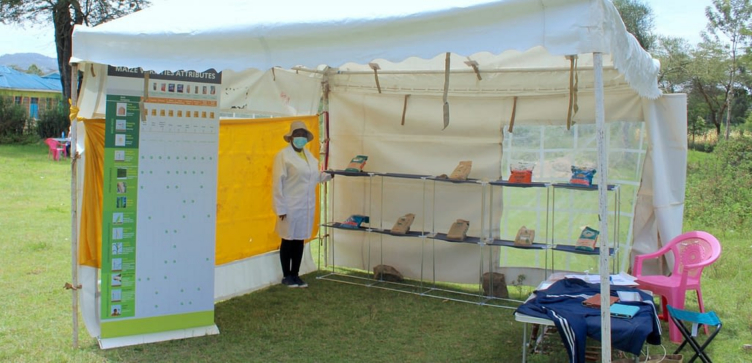
Example of mock agro-dealer store.

A mock agro-dealer store was erected in each selected village. This mock store consisted of a three square meter tent where nine commercially available, branded hybrid maize seed products were presented on shelves (Fig. 2). Products were situated on shelves each day to ensure that each appeared in the top, middle, and bottom row at least once, and also on each side and the middle of the display. Once these conditions had been met, assignment of products among the remaining placement options was randomly determined. An overview of the traits of the products offered as promoted by the seed companies is provided in Table 1. Products were selected in collaboration with seed companies active in the study area from among those currently available in the local market and suitable for the agro-ecological zone (dry mid-altitude). Two varieties per brand were offered for three companies (SeedCo, Bayer and Kenya Seed Company) and for three other companies only one product was offered through the study (Western Seeds, Dryland Seeds and Corteva).^4^ The oldest product was released 26 years ago (1995) and the most recent six years ago (2015). Two products had a lower cost than the others (DH02 and DH04, both from Kenya Seed Company) as indicated in Table 1, which is in line with market prices and the parastatal's pricing strategy. We refer to those products as ‘low-cost’, which is different than the promoted product.

**Table 1:** Maize seed products included in the study.

Product	Company	Company category	Year released	Maturity level	Estimated market share^a^	Market price (KES)	Reported yield potential (90 kg bags)	Reported Stress tolerance	Reported Agronomic/postharvest attributes
Duma 43	Seed Co	Regional	2003	Early	40%	500	30–32	Drought, cob rot, gray leaf spot and northern leaf blight	High shelling percentage, hard dent grain texture, very white grain color
Sungura 301	Seed Co	Regional	2015	Very early	7%	500	25–30	Drought, gray leaf spot	Good husk/tip cover, semi-flint grain texture, high shelling percentage
DK8031	Bayer	Multinational	2003	Early	17%	500	28–32	Drought, Maize Streak Virus, Gray leaf spot. Ear rot	Excellent milling qualities, not sensitive to planting dates, hard-dent grain
DK8033	Bayer	Multinational	2004	Intermediate	1%	500	34–36	Drought, Leaf diseases: Gray Leaf Spot, MSV, rust	Good standability, hard dent grains, double cobbing
DH02	Kenya Seed Company	Parastatal	1995	Very early	8%	400	18	Drought, Maize Streak Virus	Plant stays green
DH04	Kenya Seed Company	Parastatal	2001	Early	4%	400	24	Drought, Leaf diseases: Leaf blight and leaf blight	Good husk cover, good standability
PHB 3253	Pioneer	Multinational	1996	Intermediate	15%	500	40	Drought, Leaf diseases: Leaf blight and rust	Good standability, white and hard flint kernel
SAWA	Dryland seed Company	Local	2015	Very early	3%	500	32	Drought, Leaf diseases: Leaf Spot and Maize Streak Virus	Good husk cover, semi-dent white grain, stay green trait, sweet taste
WH101	Western seed Company	Local	2006^b^	Early	0%	500	20-25	Drought, Leaf diseases: Gray Leaf Spot, Maize Streak Virus and Blight	Suitable for 2nd season planting, sweet grain, excellent for roasting

a Market share of maize seed products in Machakos county in 2021 based on agro-dealer panel data (Rutsaert et al., 2023).

b Although WH101 was released in 2006, it was not made available on the market till 2019.

Each participating farmer was asked to visit the mock store and purchase one of the available products. Participants were randomly allocated to one of six different cells (Table 2). In half of the cells (cell 1, 3 and 5), participants had access to all products that were available in the store. Participants in the remaining cells (cell 2, 4 and 6) faced an OOS condition in which the product they had planned to purchase that season (elicited prior to their entry to the shop) was unavailable. The OOS condition mimicked a situation that farmers would face if a seed company were to replace an older product.

**Table 2:** Mock store design treatments.

	Preferred product available	Preferred product out-of-stock
Control	Cell 1	Cell 2
Information: A poster is included in the store with technical information about the available varieties	Cell 3	Cell 4
Promotion: two randomly selected products have a price promotion of 10%.	Cell 5	Cell 6

The participants in the full stock and OOS conditions were assigned to two marketing treatments:

- Control (cells 1 and 2)
- Information treatment (cells 3 and 4): a poster was put in the store with detailed information about each product taken from marketing materials shared by seed companies (Appendix Fig. 1).
- Promotion treatment (cells 5 and 6): There was a price discount of 50 KES (approximately 10% of the standard retail price) on two randomly selected products.^5^

### 3.3 Data collection protocol

Data was collected over the course of a single day in each of the 20 study villages. The number of farmers participating per village varied between 14 and 32, with a total of 600 overall. All data per participant was collected on a single tablet that was passed through the enumerator team. On average, the mock-store visit and pre- and post-visit surveys lasted 30 min and consisted of three parts:

#### 3.3.1 Step 1 – Pre-visit survey

Research assistants administered to each participant a short survey outside of the mock store prior to seeing the store setup. This included socio-demographic information, agricultural land area, maize seed product grown during in the past season, and the maize seed product the participant planned to purchase for the upcoming maize season. In the OOS situation, the enumerator informed the store clerk of the seed product the participant had indicated they planned to purchase in the pre-entry survey, and this product, if present, was removed from those available. In case of the information or promotion treatment, the information poster and the discount tags were set up before the participant entered the store.

#### 3.3.2 Step 2 – Mock store visit

The participant was invited into the mock store by a research assistant posing as a store clerk and asked to select the product they would like to purchase. Each time a product was picked from the shelves, it was replaced before the next participant entered the store. Every farmer was asked to make a choice and there was no opt-out option. This reflected the fact that all farmers recruited into the study were hybrid seed users and were expected to ultimately choose from among the product options available for commercial purchase; indeed only 1% of farmers reported storing any maize to use as seed the following season (see Table 3). The vast majority of commercially available options were offered through the experiment, except in the OOS condition, in which the choice set was deliberately constrained to mimic a situation in which companies have retired older products.^6^

**Table 3:** Participant characteristics.

	Full Sample			Control		OOS		Comparison
	Mean	SD	N	Mean	N	Mean	N	p-value
Respondent is male	0.43	0.50	597	0.43	299	0.44	298	0.841
Respondent age	49.75	14.85	597	49.61	299	49.9	298	0.813
Respondent is married	0.84	0.37	597	0.85	299	0.83	298	0.565
Education: None	0.02	0.14	597	0.02	299	0.02	298	0.564
Education: Primary	0.47	0.50	597	0.44	299	0.49	298	0.307
Education: Secondary	0.42	0.49	597	0.45	299	0.38	298	0.104
Education: Tertiary	0.07	0.25	597	0.05	299	0.08	298	0.243
Education: Other	0.03	0.18	597	0.03	299	0.04	298	0.644
Total area managed (Acres)	2.32	2.36	597	2.30	299	2.34	298	0.839
Area under maize (Acres)	1.68	1.65	597	1.71	299	1.64	298	0.630
Main product: Duma 43	0.71	0.46	597	0.70	299	0.71	298	0.673
Stored any seed for next season	0.01	0.08	597	0.00	299	0.01	298	0.315
Share maize sold	26.64	24.36	597	25.17	299	28.12	298	0.139
Share maize consumed	55.64	26.27	597	56.94	299	54.33	298	0.225
Share income from agriculture	62.09	26.83	597	62.71	299	61.46	298	0.570

Aside from the seed choice, the following other indicators were tracked to understand participants' decision process. First, a stopwatch was used to record *seed selection time*, from the moment the participant was asked to make a purchase until the final choice was made. Second, the number and type of *questions asked in the store* by participants were recorded. Third, the *level of attention* given to the seed offer was assessed by the store clerk on a observational scale ranging from 1 ‘no attention was paid to the maize seed products in the store’ to 5 ‘the participant had a detailed look at all the products in the store’.

#### 3.3.3 Step 3 – Post-visit survey

After completing the seed purchase, each participant was guided to another research assistant and administered a post-purchase survey. This survey focused on participants' reasons for choosing the seed product they had selected, recollection of the products in the store, and their knowledge and previous usage of each of these products.

### 3.4 Statistical analysis

To estimate the effect of each treatment on participants' attention to the products offered, their time spent deciding, and an indicator for whether they asked the agro-dealer any questions about products prior to purchase, we estimate the following linear regression model:

(1) $${\mathrm{PurchaseAction}}_{i,j}=\alpha +\beta_{I}{\mathrm{Information}}_{i,j}+\beta_{P}{\mathrm{Promotions}}_{i,j}+\beta_{\mathrm{OOS}}{\mathrm{OutOfStock}}_{i,j}+\varepsilon_{i,j}$$

Where *Information*, *Promotions*, and *OutOfStock* are binary variables indicating assignment of individual *i* from village *j* to each of these treatments, *PurchaseAction* indicates the outcome of interest, and ε is a stochastic error term. Results are estimated using ordinary least squares unless otherwise noted, and standard errors are clustered at the village level. We estimate the same model with interactions between the OOS condition and each of the information and promotions treatments:

(2) $${\mathrm{PurchaseAction}}_{i,j}=\alpha +\beta_{I}{\mathrm{Information}}_{i,j}+\beta_{P}{\mathrm{Promotions}}_{i,j}+\beta_{\mathrm{OOS}}{\mathrm{OutOfStock}}_{i,j}+\beta_{I\cdot \mathrm{OOS}}\mathrm{Information}\;X\;{\mathrm{OutOfStock}}_{i,j}+\beta_{P\cdot \mathrm{OOS}}\mathrm{Promot}i\mathrm{ons}\;X\;{\mathrm{OutOfStock}}_{i,j}+\varepsilon_{i,j}$$

Next, we estimate a discrete choice model using a conditional logistic regression (McFadden, 1973). Here observations are defined at the individual (*i*) by product (*k*) level (i.e., 600 individuals × 9 varieties) with a binary outcome variable indicating whether the individual chose to purchase product *k*. Standard errors are clustered at the individual level. Since most individuals not assigned to the OOS condition opted for their planned product, we estimate this model separately for the OOS (3) and control (4) conditions, with a control for planned product in the latter. We then expand these models to include a vector of additional product characteristics ($\bar{x})$ and participant-product characteristics ($\bar{z})$, such as whether the participant had ever grown the product.

(3) $${\mathrm{ChoseVariety}}_{i,k}=\beta_{\mathrm{PROM}}{\mathrm{IsPromotedVariety}}_{i,k}\;\left(+\beta_{x}\bar{x}\right)+\varepsilon_{i,k}$$

(4) $${\mathrm{ChoseVariety}}_{i,k}=\beta_{\mathrm{PROM}}{\mathrm{IsPromotedProduct}}_{i,k}+\beta_{\mathrm{PLAN}}{\mathrm{IsPlannedProduct}}_{i,k}\;\left(+\beta_{x}\bar{x}+\beta_{z}\bar{z}\right)+\varepsilon_{i,k}$$

Note that in contrast to Eqs. (1), (2), the explanatory variables in models (3) and (4) aside from *IsPromotedProduct* are not exogenously determined. The coefficients on these variables should thus be interpreted as associations rather than causal effects. For example, if we were to observe a statistically significant level of switching to low-price products when consumers' preferred product is unavailable, this would not necessarily imply that switching was a result of the lower price, as other characteristics of low-price products could be the reason for this pattern.

## 4 Results

### 4.1 Sample description and balance

The initial sample comprised 600 farmers: 200 farmers were assigned to receive the information treatment, 200 to receive the promotions treatment and 200 to the control group. Orthogonal to these treatments, 300 farmers were assigned to the cross-cutting OOS condition, and 300 to the control condition. Of the selected farmers,

two either did not know or refused to state a planned seed product, and one in the OOS treatment was able to purchase their planned brand due to an implementation error. As a result, the final analysis sample consists of 597 individuals.

Table 3 summarizes the characteristics of farmers who participated in the study and presents *p*-values for tests across the control versus OOS treatments. The average age of participants was 50 years. Most participants were female (57%) and typically married (84%). In terms of education, 47% had completed primary school, 42% had completed secondary school and 7% had completed tertiary education (2% report no education).

Farmers managed an average area of 2.32 acres of cropland, of which 1.68 acres (72%) was under maize, though there is a high degree of variation in both measures, with farmers reporting between 0.25 and 25 acres of total cultivated land and 0.13–18 acres under maize. The average farmer reported using more than half of their harvested maize crop (56%) for household consumption and selling slightly more than a quarter (27%) in the market (with the remaining harvested quantity used for other purposes or lost prior to sale or consumption). However, we observe that some farmers grew exclusively for household consumption while others were fully commercialized. By far, the most cultivated maize seed product was Duma 43, grown by 71% of participants in the most recent season.

These characteristics were balanced across treatment groups. Farmers assigned to the out-of-stock treatment were comparable to those in the control group, with no statistically significant differences observed between groups. Likewise, farmers assigned to the information or promotions treatments were similar to those who were not assigned to either (Appendix Table 1).

### 4.2 Behavior in the retail environment

Table 4 summarizes the percentages of farmers in the OOS and control conditions who selected products in terms of their stated plan, the product's promotion status, brand, relative price, experience, and knowledge. For control farmers, seed choice was dominated by the product which they reported they planned to purchase in the pre-visit survey: 88% of non-OOS farmers overall (and 98% of non-OOS farmers whose planned product was available), went on to purchase that product. An additional 3.3% purchased a different product from the same brand. Among individuals in the OOS treatment (who were unable to purchase their planned product),^7^ one third chose a product of the same brand as their planned product (such a product was available for 95% of these participants). Over 90% of control group farmers chose a product which they had previously grown, relative to 52% in the OOS situation. Low-cost products were not popular choices among either group, but participants assigned to the OOS condition were more likely to choose one of the products promoted through a price discount relative to the availability of such products.

**Table 4:** Seed choice, by OOS treatment status.

	Control		OOS	
	Chosen	Available products meeting criterion	Chosen	Available products meeting criterion
Planned product	88.3%	10.9%	0.0%	0.0%
Promoted product	7.7%	7.4%	11.1%	7.6%
Other product, same brand	3.3%	10.4%	32.9%	12.1%
Low-cost product	8.7%	22.2%	14.1%	24.2%
Product previously grown	92.3%	30.5%	52.4%	23.4%
Product seen growing, but not grown	3.7%	18.2%	19.8%	21.8%
Product heard of, but not seen	3.0%	26.5%	19.5%	26.7%
Observations	299	299	298	298

“Low-cost product” refers to the two products which were priced 20% lower than the other seven available.

Table 5 shows the products farmers planned to buy before entering the mock store and those that they actually purchased, both in the control condition as the OOS condition. As indicated in the sample description, Duma 43 was the product that most farmers planned to buy and actually purchased in the control condition. Of the 207 farmers in the OOS condition who planned to purchase Duma 43, 59 switched to a different product from the same brand, Sungura 301. They also switched to other products including those from Bayer, 45 farmers went for DK8031 and 42 farmers switched to DK8033, and PHB3235 of Corteva, selected by 26 farmers. Brand loyalty was stronger among the farmers who planned to purchase Bayer products: 22 of the 38 farmers planning to buy DK8031 switched to DK8033 and 8 of the 17 farmers planning to buy DK8033 switched to DK8031, thereby capturing 55% of their customers in case of OOS. Haraka 101 and SAWA, both launched by local seed companies and only recently made available on the market, were unsuccessful in attracting farmers in the control condition nor the OOS condition.

**Table 5:** Crosstab between the products farmers planned to buy and the products they purchased.

Control condition (n = 299)											
		Product farmer bought									
		Duma 43 SeedCo	Sungura 301 SeedCo	DK8031 Bayer	DK8033 Bayer	DH02 KSC	DH04 KSC	PHB3253 Corteva	SAWA Dryland Seed	Haraka 101 Western Seed	Sum
Product farmer planned to buy	Duma 43	64.2%	2.0%	0.3%	0.7%	0.7%		1.7%	0.3%	0.3%	70.2%
	Sungura 301		1.0%								1.0%
	DK8031	1.3%		6.4%			0.3%				8.4%
	DK8033	0.3%		1.0%	4.7%						6.0%
	DH02					6.0%					6.0%
	DH04						1.7%				1.7%
	PHB3253	0.3%						4.3%			4.7%
	Other	0.3%	0.3%	0.3%				1.0%			2.0%
	Sum	66.6%	3.3%	8.0%	5.7%	6.7%	2.0%	7.0%	0.3%	0.3%	


Out-of-stock condition (n = 298)											
		Product farmer bought									
		Duma 43 SeedCo	Sungura 301 SeedCo	DK8031 Bayer	DK8033 Bayer	DH02 KSC	DH04 KSC	PHB3253 Corteva	SAWA Dryland Seed	Haraka 101 Western Seed	Sum
Product farmer planned to buy	Duma 43		19.8%	15.1%	14.1%	6.0%	4.7%	8.7%		1.0%	69.5%
	Sungura 301	0.3%		0.7%							1.0%
	DK8031	4.0%	0.3%		7.4%		0.3%	0.3%			12.8%
	DK8033	2.7%	0.3%	2.7%							5.7%
	DH02	0.7%	0.3%	1.3%			2.7%		0.3%	0.3%	5.7%
	DH04	0.7%									0.7%
	PHB3253	2.7%		0.3%			0.3%				3.4%
	Other	1.0%			0.3%			0.3%			1.7%
	Sum	12.1%	20.8%	20.5%	21.8%	6.0%	8.1%	9.4%	0.3%	1.3%	

Table 6 presents the effects of each of the treatments on behavior in the retail environment, based on the regression specifications described in Eqs. (1), (2). Outcomes are variables indicating whether the participant paid significant attention to the varieties on offer; the amount of time spent making their choice (in seconds); and whether they asked any questions about varietal traits. Odd-numbered specifications report the results of estimating Eq. (1), while even-numbered specifications include interactions between the cross-randomized treatments as shown in Eq. (2).

**Table 6:** Treatments effects on behavior in the retail environment.

	Paid significant attention		Time on choice (seconds)		Asked about varieties	
	(1)	(2)	(3)	(4)	(5)	(6)
OOS	0.38***	0.40***	42.76***	31.33***	0.18***	0.13***
	(0.04)	(0.06)	(4.35)	(6.15)	(0.02)	(0.04)
Information	0.04	0.08	9.71**	1.96	0.03	0.00
	(0.05)	(0.07)	(3.78)	(2.86)	(0.03)	(0.02)
Info x OOS		−0.08		15.53		0.06
		(0.08)		(10.06)		(0.06)
Info + Info x OOS		0.00		17.49		0.06
		(0.07)		(8.49)		(0.06)
Promotions	0.06	0.04	7.73	−1.61	0.03	−0.02
	(0.04)	(0.05)	(4.79)	(3.07)	(0.03)	(0.01)
Promo x OOS		0.03		18.78		0.09
		(0.08)		(12.00)		(0.07)
Promo + Promo x OOS		0.07		17.17		0.07
		(0.06)		(10.46)		(0.07)
Control mean	0.20	0.20	35.23	35.23	0.02	0.02
Control SE	0	0	2	2	0	0
Observations	597	597	597	597	597	597

Ordinary least squares regression of treatment status on behavior in the retail environment. Standard errors are clustered at the village level. *,**,*** indicate significance at the 10%, 5% and 1% levels respectively.

We observe a consistently positive and statistically significant relationship between assignment to the OOS condition and each of these purchase behavior outcomes. Participants assigned to the OOS condition paid more attention to the products offered than those who had the option of selecting their preferred product (column 1). The modal participant in the control group was observed to scan over products with very little engagement, while the modal participant in the OOS treatment had a careful look at the products available. Those in the OOS condition spent considerably longer making their choice: approximately one minute and eighteen seconds, versus 35 s in the control group (column 3). They were also far more likely to ask questions: 20% of those in the OOS condition asked a question about product attributes, relative to only 2% in the control group (column 5).

There is also a positive and significant effect of assignment to the information treatment on the time spent on choosing a product in the pooled (control and OOS) sample (column 3). Results shown in column 4 reveal that this effect is driven by participants assigned to both the OOS and the information treatment, though the treatment interaction term is not statistically significant. The point estimate of the impact of promoting certain varieties through a 10% discount in both the pooled sample and within the OOS condition is consistently positive but never significantly different from zero.

Considering heterogeneity in treatment outcomes by gender, we find a stronger effect of the OOS treatment among female participants on the amount of attention paid to the varieties available (Table 7). We also observe that the effect of the promotions treatment on time spent on choice is driven entirely by male respondents. We find no differences in the effect of the information treatment by participant gender.

**Table 7:** Heterogeneous effects of treatments on behavior in the retail environment by gender.

	Paid significant attention	Time on choice (seconds)	Asked about varieties
	(1)	(2)	(3)
Information	0.115	11.937	0.036
	(0.081)	(7.664)	(0.051)
Promotion	0.123*	18.398**	0.044
	(0.065)	(8.480)	(0.046)
OOS	0.286***	45.359***	0.153***
	(0.052)	(7.425)	(0.038)
Is female	−0.032	5.366	−0.017
	(0.075)	(5.802)	(0.040)
Info x Female	−0.124	−3.975	−0.011
	(0.125)	(9.398)	(0.066)
Promo x Female	−0.119*	−18.991**	−0.033
	(0.068)	(8.708)	(0.055)
OOS x Female	0.164**	−4.724	0.050
	(0.059)	(8.916)	(0.039)
Info +			
Info x Female	−0.01	7.96	0.02
	(0.08)	(4.54)	(0.04)
Promo +			
Promo x Female	0.00	−0.59	0.01
	(0.04)	(4.28)	(0.04)
Control mean	0.20	35.23	0.02
Observations	597	597	597

Linear probability regression models of treatment status and gender on behavior in the retail environment. Standard errors are clustered at the village level. *,**,*** indicate significance at the 10%, 5% and 1% levels respectively.

### 4.3 Product choice

**Fig. 3:**
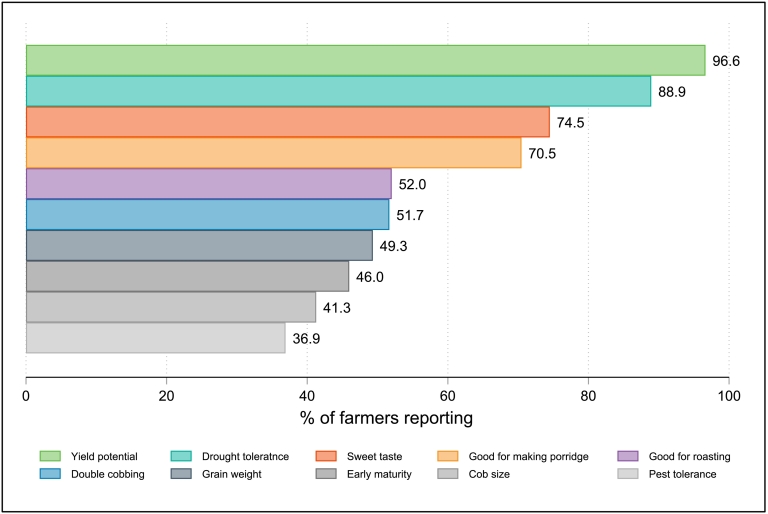
Reported preferences influencing product selection, non-OOS control sample.

We next look at factors correlated with product choice. Fig. 3 presents the product attributes reported by participants in the OOS control group as most important for determining their choice of maize seed product during the experiment. As the figure shows, yield and drought tolerance are first order concerns for farmers in the sample, reported by 98% and 90% respectively as one of the most important traits influencing their selection. Perhaps unsurprisingly given the high levels of auto-consumption, palatability is also very important, with 76% of farmers identifying “sweet taste” and 70% reporting good properties for making maize porridge (a primary staple in Kenya) as among the most desirable traits. Agronomic features (early maturity, double-cobbing, ease of forecasting, pest tolerance and cob size) were also mentioned by some farmers but were less likely to be reported as determinant of seed product choice.

To explore the relative importance of the price promotion treatment, the farmer's original intention, brand, price, experience, and knowledge, we regress an indicator variable for whether the product was selected on the product-specific treatment indicator (*Promotions*), and an indicator for whether the product purchased was the planned product, following the specifications described in Eqs. (3), (4). To these we add additional explanatory variables for whether the product was an alternative within the same brand as the planned product, whether the product was low-cost, and the participant's experience and familiarity with the product. With nine products offered per farmer in the control group and 8 in the OOS situation, the total sample size is 299 × 9 = 2691 and 298 × 8 = 2389 for the control and OOS specifications respectively.

Estimated odds ratios of seed choice are presented in Table 8. The first two columns show results from the parsimonious specification (3), in which only indicators for the randomly varied price promotion treatment and (for the non-OOS sample) planned product are included. Columns 3 and 4 include additional controls for non-experimental factors influencing seed choice (specification 4). When considering the influence of product attributes, our preferred specification for the OOS sample, shown in column 5, includes a control for whether an alternative product within the same brand as the one the farmer planned to purchase was available. As noted above, this was the case for 95% of farmers, hence we do not estimate this equation for the OOS control group.

**Table 8:** Predictors of product choice, by out-of-stock treatment status.

	Control	OOS	Control	OOS	OOS
	(1)	(2)	(3)	(4)	(5)
Promoted product	1.10	1.76***	1.03	1.73**	1.66**
	(0.44)	(0.38)	(0.42)	(0.40)	(0.39)
Planned product	72.85***		39.36***		
	(14.29)		(10.84)		
Alternative product within planned brand			4.12***	4.89***	5.38***
			(1.63)	(0.76)	(1.59)
Low-cost product			0.91	0.69**	0.26
			(0.24)	(0.13)	(0.29)
Ever grown			9.34***	14.97***	8.11***
			(5.54)	(4.07)	(2.84)
Ever seen			2.17	3.67***	2.21**
			(1.41)	(1.02)	(0.75)
Ever heard of			2.04	2.18***	1.38
			(1.33)	(0.63)	(0.46)
Brand control	No	No	No	No	Yes
Individuals	299	298	299	298	298
Observations	2691	2389	2691	2389	2389

Conditional logistic regression of seed product attributes on seed choice. Coefficients are odds ratios. Standard errors in parentheses are clustered at the individual level. *,**,*** indicate significance at the 10%, 5% and 1% levels respectively. Observations are at the farmer-product level, with nine varieties per farmer. “Low-cost product” refers to the two products that were priced 20% lower than the other seven available. “Brand control” refers to the inclusion of an individual-level control for whether an alternative product within the same brand was available.

Confirming the descriptive results shown in Table 4, the most influential factor associated with seed choice for farmers in the control (non-OOS) condition was reported planned product. The randomly varied price promotion had no impact on the choices of farmers who could purchase their planned product.

However, when this was not an option, promotion increased the odds of product selection by between 76% (column 2) in the specification without controls for other factors influencing this choice, and 66% when these are included (column 5). As noted above, the impact of the promotion treatment appears, based on participants' behavior in the retail environment, to be driven by male farmers (Appendix Table 1).

Farmers' previous experience growing a product was the most influential common predictor of product choice across the two conditions, increasing the odds of purchase by nine times in the control condition and eight times in the OOS condition (columns 3 and 5). Brand was also important, with the odds of purchasing a product from the same brand as the planned product four times higher than those of purchasing one sold under a different brand in the control condition (column 3), and five times higher when the preferred brand was unavailable (column 5). Having seen a product growing doubled the odds of selection in the OOS condition, but simply having heard about it had no effect (column 5).

## 5 Discussion

### 5.1 In-stock vs out-of-stock

This study examined Kenyan farmers' maize seed product choice at the point-of-purchase and the factors which might influence that choice. When farmers had access to their preferred product (i.e., the product they planned to purchase prior to entering the mock store), the speed of product selection was remarkably fast despite the range of products available from which to choose. Farmers generally ignored information about the seed products and the ten-percent promotional discounts. These results are consistent with previous findings on customer behavior in the context of repeatedly purchased products (Anesbury et al., 2016; Hoyer, 1984).

When farmers did not have access to their preferred products (ie, when we created an OOS condition for half the sample), the experiment simulated the choice scenario faced by farmers when seed companies replace an older seed product. In theory, this situation would push farmers to reflect over product selection, possibly influenced by in-store environmental cues (e.g. price promotions, information). Even in the OOS condition, however, the effects of both the poster and the price promotions were limited. The main influencing factor for picking a product was participants' previous experience with a product, and secondly, the brand. However, most farmers stated that seed product attributes were critical to their selection decision and that they based their choice on attributes such as yield, drought tolerance and taste. That previous experience and brand loyalty emerged as the two most important predicators of farmers' selection in an OOS condition suggests a need to revisit basic assumptions that underpin how hybrid maize seed systems operate, e.g., farmers are willing substitute existing products for products with higher yield potential.

So why was it that seed product choices are made in such a fast and decisive way? In the following two subsections, we reflect on the store environment and different influencing factors farmers encounter in the store. In the last subsection, we discuss the experimental design of the study and some limitations.

### 5.2 Information on seed attributes

In theory, reliable third-party information on seed product performance should address the unobservable nature of seed quality. Without access to accurate information on seed performance, farmers resort to observations of seeds grown by their peers and the marketing efforts of seed companies.

The notion that farmers select products based on their performance along dimensions they value (expected yield, tolerance of extreme weather conditions) has been a central theme in explaining the uptake of maize hybrids in the United States, both during the early stages of hybrid deployment (Griliches, 1957) and more recently with the uptake of drought tolerant hybrids (McFadden et al., 2019). As noted by Duvick and Cassman (1999) in the case of US maize farmers' uptake of new maize hybrids: “Farmers also show little company loyalty, if it looks like ‘loyalty’ is going to cost them money.” On-farm performance of new maize hybrids released in the United States is reported by seed companies across multiple criteria in data-rich publicly available reports, based on testing that involves tens of thousands of farmers over multiple years (Cooper et al., 2014; Gaffney et al., 2015; Troyer, 1996). Seed companies also pay to participate in university-sponsored performance trails where the data and analysis are made public (e.g. Kohn et al., 2021). Grassroots testing networks such as the US Testing Network have also emerged to aggregate on-farm testing data under particular growing conditions, such as organic management (US Testing Network, 2018).

However, such information does not exist in Kenya, nor if it did exist, would we expect farmers to respond in a manner similar to the US example which we just described. While new varieties are tested during two seasons of National Performance Trials (NPTs) by the Kenya Plant Health Inspectorate Service (KEPHIS) before release, the results of these trials are confidential and are not made available to farmers, retailers or seed companies. Information available through the National Crop Variety List (www.kephis.org) consists of selected traits without indications of performance relative to other varieties. For a subset of varieties (e.g., those advanced by CIMMYT and other research organizations) information on performance can be found in academic journals (e.g. Rezende et al., 2020; Worku et al., 2020) but this is generally not accessible to farmers and retailers. In the absence of effective extension programs (Tata and McNamara, 2018), farmers have little or no access to objective information to guide their decisions. One likely partial explanation is that farmers lacked confidence in the brand to decide whether to test the hybrid in their maize field. Moreover, local seed companies are reluctant to invest in building the capacities of retailers to better market seed products (reflecting, in part, the short survival period of many agro-dealers), nor do they engage with farmers during the seed selling season inside the retail space.

The overall lack of reliable data on the on-farm performance of new cereal products has been recognized and, at times, fueled intense discussion (Orr et al., 2008; Wopereis et al., 2008). Tripp (2006, p.9) argued “if researchers hope to offer farmers a range of new varieties, more investment is needed to disseminate information about these varieties. Currently, farmers depend mostly on other farmers for information about new varieties.” Farmers' access to reliable information on the performance of new products is key to informing seed choice. However, in the absence of such information to farmers and acknowledging the experience and credence attributes of seed, one could compare farmer seed choice to visiting a supermarket in a country where one does not speak the language, i.e., lots of product choice but little or no guidance on which product best meets their requirements.

Although farmers explained their product choice based on specific product attributes they required, primarily yield potential and drought tolerance, the presence of a poster providing this information for the products available had no effect on their behavior when their preferred product was available. Even in the OOS condition, 76% of farmers paid no attention to the information provided, while 11% scanned over this information. Only 13% of the farmers in an OOS condition were observed to review the poster to some degree. Should quality information on product performance become available, it will thus be important to consider how best to communicate this to farmers. In the current study, information on products was provided in a single format (i.e., a poster that compared all available varieties across many traits). Additional research through which the content (e.g., detailed varietal information vs. ranking of varieties), source (e.g., non-verbal, extension officer, agro-dealer, seed company representative) and format (e.g., poster, video, audio, verbal) of information is varied could potentially identify more successful ways of informing farmers.

However, more reliable information alone might not be sufficient to nudge farmers' purchase behavior. Farmers may have also showed limited interested in exploring new, or otherwise unfamiliar, seed products if they were convinced that new products, in general, would not meet their expectations. New hybrid maize seed products are designed to provide incremental yield improvements over previous-generation products. In this sense, larger-scale, mechanized farmers who produce under irrigated conditions have an incentive seek out the highest performing new maize seed products. However, these products may be of limited interest to smallholders whose productivity levels under rainfed conditions fall way below the yield potential of older generation seed products (van Ittersum et al., 2016). New products may fail to deliver on farmers' expectations related to taste, storage, flour conversion, or other consumption related traits. The maize seed product H614D, launched by Kenya Seed Company in the 1980s, quickly became one of the most popular products in Kenya and has maintained a significant market share well into the present (de Groote et al., 2002; Hassan et al., 1998; Rutsaert and Donovan, 2020b; Smale and Olwande, 2014). Christinck et al. (2018) reported that farmers mostly appreciated H614D for its harder grains that are resistant to mold and insect damage, making it good for storage, as well as the flavor of the grain which was very well suited for maize porridge. Economists have recognized the importance of seed product attributes in addition to yield, and through choice experiments and other methods have sought to prioritize trait preferences (e.g. Maligalig et al., 2021; Marenya et al., 2021; Steinke and van Etten, 2017)). Important work lies ahead to obtain a better understanding the set of essential traits that seed products must have to meet the expectations of smallholders.

Our findings support the idea that buyers use brand to assess credence and experience attributes of seed products. In the OOS condition, brand was the second most important factor that guided product choice, following previous experience growing the product. A well-established company would hesitate before damaging its brand reputation by offering low-quality products or a new product that has not been properly tested over a range of agro-ecological conditions (van Etten et al., 2019). The large role played by brand loyalty in driving farmers' product selection conveys advantages to larger-scale seed companies able to invest heavily to maintain brand loyalty for existing products or build brand recognition for newly launched products. For smaller-scale seed companies, however, the investments required to replace older products that continue to enjoy strong brand loyalty are likely to be prohibitive. For decades donor support has been channeled to local seed companies in Eastern Africa to support their capacity to design and multiply seed products (AGRA, 2017). Such support, however, has not been available to support seed companies and retailers to drive the sales of new seed products.

The strong reliance by farmers on brand and previous experience for the selection of seed products poses certain challenges to policy proposals that would force seed companies to retire or deregister outdated products (Spielman and Smale, 2017). ‘Forced retirement’ policies, by design, would reduce the average weighted age of hybrid maize seed products in the market, but might also stall maize seed systems development over the long run. If newer products were higher priced or lacked certain attributes (traits) that are preferred in older products (e.g. better storage and taste), would farmers return to seed saving practices? Both farmers and retailers would lack the information required to select the ‘right’ replacement product, thus potentially favoring larger-scale seed companies with the marketing budgets able to build brand recognition. Given these unknowns, seed systems development goals might be better served through demand-side interventions related to reliable information on seed performance, seed business and retailer incentives, and consumer culture change around seed experimentation.

### 5.3 Pricing

Research has noted the potential for cost to limit the uptake of hybrid maize seed products (Simtowe et al., 2020; Smale and Olwande, 2014), especially for women farmers (Adam et al., 2020). Price promotions can be used to influence choice by manipulating this important search attribute at the point of sale. In general, the high costs of inputs poses a challenge for smallholders and especially women to intensify their on-farm production (Peterman et al., 2014). Overall, however, the effect of a small price promotion of 10 % treatment on both seed selection behavior and varietal choice in the OOS condition was limited. The limited impact of this treatment could be due to the relatively low magnitude of the discount. Given the low margins on hybrid maize seed products (Rutsaert et al., 2021), it is doubtful that cash-strapped local seed companies or agro-dealers would be able to offer deeper discounts. Small price promotions without additional information on seed performance may therefore have limited potential – at least within the commercial context – to influence farmers' seed choices. A higher level of promotion (e.g. provided through public-provided subsidies), for example, may induce more farmers to respond to the promotion. Promotions in our experiment were randomly assigned to two products in the mock store, without a specific reasoning for giving those products a promotion. That might have also contributed to low response rates.

Contrary to expectations based on previous research showing higher price sensitivity among women, the price promotion had no impact on women farmers. One possible reason could be that female farmers in our sample were more risk averse and less willing to try something new (Magnan et al., 2020). Another reason could be that women felt less permitted to experiment with unknown varieties due to decision-making power in the household regarding varietal selection (Acosta et al., 2020; de Brauw, 2015). As discussed by Voss et al. (2023), there is a high degree of joint decision-making with regards to maize farming but men tend to exert more control over seed and other input purchase decisions.

In general, the use of price promotions for agricultural inputs with strong credence and delayed experience attributes merits discussion. Much of what we know about the marketing of experience and credence goods comes from the Global North for non-agricultural products. Where consumers are unable to assess the quality of a product, price provides a critical extrinsic signal of quality. If the price of a product is reduced, the consumer's perceived financial risk for experimentation will diminish as the cost of the product does. However, consumers may perceive that more expensive products have higher quality than lower-priced products (Levin and Johnson, 1984). A retailer that discounts a product's price by offering discounts (coupons, rebates) could inadvertently lead consumers to think the product is of poor quality in case the reason for promotion is not properly communicated. In other words, discounts may invoke a trade-off between price and perceived quality. The mock store featured two products by Kenya Seed Company that had a lower (non-discounted) price relative to the other products. In the OOS, this did not trigger farmers to prioritize these products. Thus far, however, little research has been done in this area in the Global South, and no research has been carried out in the context of seed while price setting is a critical part of a formal seed system targeting smallholder farmers.

### 5.4 Reflections on the experimental design

By using a mock store we were able to control factors which would have been uncontrollable in a real-world retail setting. Foremost was the OOS condition: one can easily imagine the unenthusiastic responses by shop owners at the prospect of removing their best-selling products for the purposes of research. Secondly, our goal was to monitor farmers when they made their seed product choice. To have done this in an actual store would have run the risk of study participants behaving differently than they normally would have. Lastly, the mock store allowed us to easily manipulate the environment (e.g. add price promotions or additional information about the seed).

Of course, our mock store did not fully recreate a real-world seed product purchase. As mentioned in the methods section, each participant was asked to make a seed choice from the available options in the shop and was not allowed to opt out, even in the OOS condition. However, as it would not be ethical to force farmers into a seed choice which they would not normally make, farmers were given an endowment to participate that was higher than the cost of the seed choices in the mock store, potentially leading to a windfall effect (Carlsson et al., 2013), i.e. different behavior with ‘given’ money than with earned money. Additionally, there could be a high probability of ‘no purchase’ in a real agro-dealer in the case of an OOS condition (Verbeke et al., 1998). The study was scheduled before the start of the rainy season so farmers had yet to purchase seed for the upcoming planting season.

Agro-dealers may have a trust relationship with their customers and look to them for guidance on their seed choice. Rutsaert and Donovan (2020b) showed that this mostly happens in cases where farmers were uncertain about want to buy, often due to their preferred product being unavailable. When farmers know which product to buy, interaction is minimal and purchases happen quick. In our mock store, interaction with the agro-dealer was mainly sought in the OOS condition, which is in line with the findings above. However, the setup of the study was not aimed to test the role of the agro-dealer in seed product choice. The agro-dealer, always played by the same research assistant, did not recommend products to participants and tried to minimize influence on the product choice. But having someone in store allowed to make observations such time spent on seed choice and attention paid to store offer.

Lastly, our results are influenced by the intensity of the two treatments used in the study (ie, 10 % randomly assigned price promotion and seed product information poster). The design of these treatments reflected our understanding of the seed industry in Kenya (e.g. the percentage of price promotion that would be agreeable to seed companies) and our thoughts on what information might be useful for farmers. Given the unique nature of this study, we were unable to rely on previous studies to guide the design of the treatments. A price promotion of 10% might have been too low to shift farmers away from their preferred choices and other more interactive (intensive) methods than a poster might be more effective to share information with farmers. To ensure enough statistical power per treatment, we did not combine the promotion and information treatment. As we wanted to exclude interaction between the promotion and a particular seed product, the choice was made to randomize the price promotion across all seed products in the study. Future research with more specific aims to promote new products should ensure that strong treatments in terms of price and/or information are used to influence farmer seed choices.

## 6 Conclusion

Within the Kenyan maize seed system most farmers purchase maize seed products on an annual basis – a condition which partially explains the strong private sector investment in seed production and distribution over multiple decades. Yet, even under these favorable conditions, persistent and critical ‘last mile’ hurdles exist to encourage farmers to purchase new seed products, thereby reducing the impact from investments in maize breeding.

This paper is one of the first to provide insights on how farmers make decisions about seed products in a limited-information environment. It highlights the need for deeper engagements between retailers, seed businesses, and farmers to drive innovation and experimentation in seed product value chains. In the absence of a reliable third-party information on hybrid maize seed product performance, increased experimentation by famers with new seed products will be key to achieve faster varietal turnover. This work builds on previous discussions on integrated seed sector development by exploring how risk aversion and seed-purchasing culture, economic incentives, and seed product information shape seed value chain relations, and ultimately, the performance of seed systems. The results of our study raise four under-explored issues for the future development of hybrid maize seed systems in East Africa:

First, viable solutions to slow varietal turnover will require interventions to nudge farmers to experiment with new seed products. Our work explored the potential for two interventions—information and price discounts—to influence farmers' product selection. While these interventions showed limited influence on selection, the study design provides a clear starting point for future related experiments.

Second, the information environment in Kenya fails to support informed product decision-making by farmers. Public and private investments will be required to generate timely, comparable, and reliable information on seed performance. Easy access to such information should increase competition among seed companies and support retailers in their own marketing efforts.

Third, the strong effect of brand loyalty likely disadvantages local, often smaller-scale, seed companies relative to their larger-sized counterparts (Donovan et al., 2022). Local seed companies tend to be major recipients of new maize varieties from public-sector breeding programs and donor investments in seed enterprise development (AGRA, 2017). Efforts to support seed marketing by local companies will require new conversations on the nature of public-private partnerships in seed systems.

Finally, the tendency for farmers, when faced with the OOS condition, to prioritize familiar seed products (ie, products with which they know from previous experience) suggests the need to better understand the potential to leverage point-of-sale to encourage product experimentation. Along the same vein, policies that would require seed companies to withdraw old seed products from the market run the risk of pushing small seed companies out of the market and encouraging farmers to save their seed.

## CRediT authorship contribution statement

**Pieter Rutsaert:** Writing – review & editing, Writing – original draft, Visualization, Supervision, Project administration, Methodology, Investigation, Conceptualization. **Jason Donovan:** Writing – review & editing, Writing – original draft, Visualization, Supervision, Resources, Project administration, Methodology, Investigation, Funding acquisition, Conceptualization. **Mike Murphy:** Writing – review & editing, Writing – original draft, Software, Formal analysis, Data curation. **Vivian Hoffmann:** Writing – review & editing, Writing – original draft, Methodology, Formal analysis, Conceptualization.

## Declaration of competing interest

None of the authors have relevant material or financial interests in the subject of the research and the authors declared no potential conflicts of interest with respect to the research, authorship, and/or publication of this article.

## Figures and Tables

**Table:** Table A1: Balance table showing *p*-values testing for differences by treatment assignment.

Control	No treatment	No treatment	No treatment	OOS only	OOS only
Treatment	OOS only	Info only	Promo only	OOS + Info	OOS + Promo
Respondent is male	0.936	0.714	0.887	0.951	0.887
Respondent age	0.667	0.287	0.831	0.974	0.896
Respondent is married	0.874	0.332	0.85	0.822	0.579
Education: None	0.712	0.417	0.176	0.644	0.082
Education: Primary	0.008	0.039	0.045	0.231	0.396
Education: Secondary	0.018	0.039	0.159	0.812	0.561
Education: Tertiary	0.988	0.148	0.702	0.063	0.236
Education: Other	0.712	0.180	1.000	0.712	0.702
Total area managed (Acres)	0.678	0.594	0.784	0.115	0.822
Area under maize (Acres)	0.369	0.876	0.789	0.063	0.946
Main product: Duma 43	0.717	0.198	0.275	0.381	0.748
Stored any seed for next season	0.321	0.321	0.319	0.159	0.319
Share maize sold	0.999	0.554	0.371	0.120	0.174
Share maize consumed	0.853	0.375	0.198	0.277	0.420
Share income from agriculture	0.644	0.693	0.133	0.386	0.626
Observations	199	199	200	199	198

**Figure:**
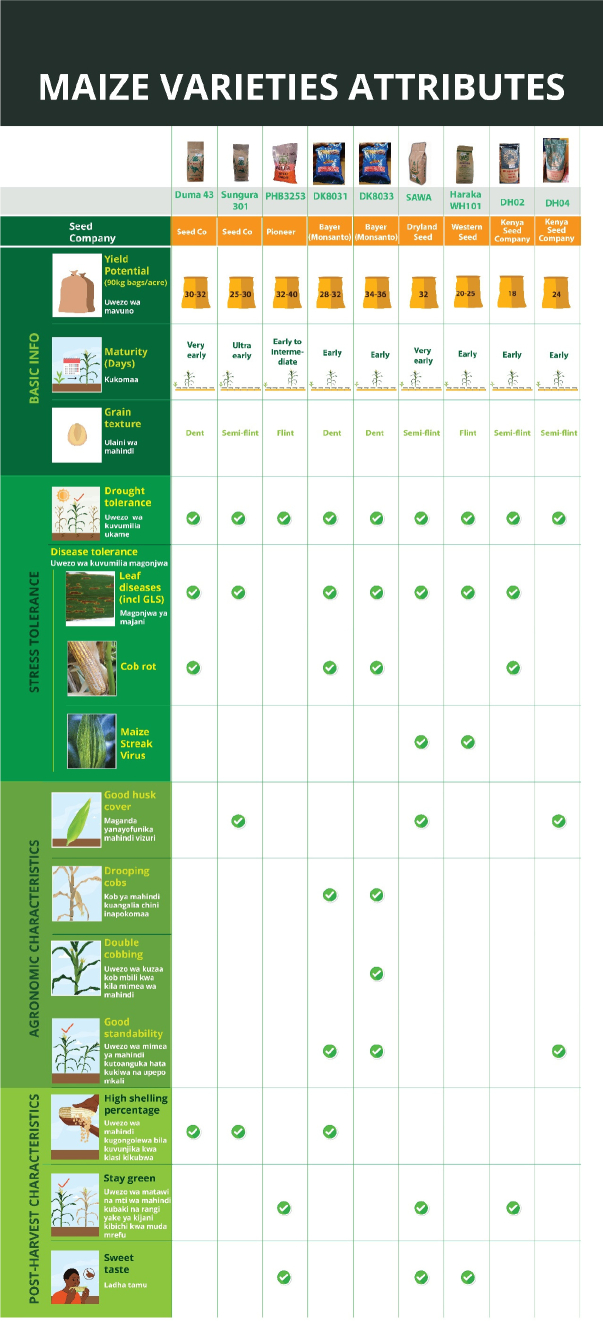
Fig. A1: Information poster about products in the mock agro-dealer.

## Data Availability

Data will be made available on request.

## Footnotes

5 The varieties that received a price promotion were randomly assigned before the start of the study. In case of the OOS cells, it was possible that one of the promoted seed products overlapped with the OOS option, resulting in only one promoted seed product being available in the store.

## Footnotes

1 Once a new hybrid (or improved variety) is released and commercially available to farmers, we consider it a seed product.

2 Ethical approval was obtained from the International Livestock Research Institute (ILRI-IREC 2021–15).

3 In villages with fewer than 16 men or fewer than 16 women farmers, additional farmers of the other gender were selected to reach the target of 32 farmers per village.

4 The original plan was to have a second Corteva product in the experiment but due to low availability and problems with sourcing during the fieldwork, this product was excluded.

6 As noted by Campbell and Erdem (2019), for studies with the primary objective of estimating marginal rates of substitution among attributes or alternatives, an opt-out may be unnecessary.

7 Due to an administrative error, one individual in the OOS treatment was able to access their planned brand. We retain this individual in the dataset. Excluding them does not meaningfully alter our findings.
